# Effect of Transformation Plasticity on the Residual Stress of Laser–MAG Hybrid Welding of 30MnCrNiMo High-Strength Steel

**DOI:** 10.3390/ma19051022

**Published:** 2026-03-06

**Authors:** Haotian Sun, Yongquan Han, Ruiqing Lang, Boyu Song, Zhenbang Sun, Xulei Bao

**Affiliations:** 1 School of Materials Science and Engineering, Inner Mongolia University of Technology, Hohhot 010051, China; 20211000038@imut.edu.cn (H.S.);; 2 Engineering Research Center of Development and Processing Protection of Advanced Light Metals, Ministry of Education, Hohhot 010051, China; 3 School of Materials Science and Engineering, Inner Mongolia University of Science & Technology, Baotou 014017, China

**Keywords:** 30MnCrNiMo, thermal–metallurgical–mechanical, weld residual stress, transformation plasticity, solid-state phase transformation

## Abstract

In the current numerical simulation study of high-strength steel welding, ignoring the phase transformation plasticity effect in the coupling analysis led to a significant deviation between the simulated value of residual stress and the experimentally measured value. To investigate the influence mechanism of the Welding Residual Stresses (WRSs) of 30MnCrNiMo armor steel, the transformation plasticity (TP) coefficient (7.81 × 10^−5^ MPa^−1^) was measured via a Gleeble 3500, and a Finite Element Model (FEM) of thermal–metallurgical–mechanical coupling considering yield strength, volumetric strain and TP behavior in Solid-State Phase Transformation (SSPT) was developed. The results show that the volume expansion during the SSPT is the main factor for the shift in WRS from tensile to compressive. In contrast, the TP effect reduces the peak longitudinal tensile stress in the Heat-Affected Zone (HAZ) by 51 MPa. It also ultimately neutralizes the compressive component in this region. When the martensite fraction ranges from 0.12 to 0.45, transformation plastic strain becomes the dominant factor, leading to a characteristic evolution of longitudinal stress that initially decreases and subsequently increases. The FEM incorporating the TP effect successfully captures the dual reversals of residual stress in the HAZ. The average relative error between the simulated longitudinal stress and the experimental data obtained via X-ray diffraction (cosα method) is 8.8%. The TP coefficient database and the developed multi-field coupling model markedly enhance the predictive accuracy for WRS in 30MnCrNiMo steel, offering a robust theoretical foundation for the design of stress corrosion resistance and the service life assessment of welded joints in armored vehicles.

## 1. Introduction

30MnCrNiMo armor high-strength steel is extensively utilized in critical load-bearing components of armored vehicles as well as in armor-clad vehicle bodies [1]. This steel undergoes martensitic transformation during welding and heat treatment processes, requiring the satisfaction of stringent strength and design requirements [2]. However, welding of high-strength steel induces substantial Weld Residual Stresses (WRSs) in the vicinity of the joints, which can adversely affect the final geometry and assembly accuracy of welded structures [3]. Moreover, in the demanding service environments faced by armored vehicles, welded joints are particularly vulnerable to stress corrosion. Consequently, a comprehensive understanding of the distribution of WRS is essential. Such knowledge is fundamental not only to the precision manufacturing of armored vehicle components but also to ensure the overall quality and operational reliability of the final product.

The establishment of computational welding mechanics has played an increasingly important role in simulating complex welding phenomena, with finite element analysis technology being widely used to analyze key structures. Lu et al. [4] investigated the hybrid welding of high-strength steel using numerical methods and analyzed variations in weld geometry and WRS under different welding conditions. Their findings indicate that Solid-State Phase Transformation (SSPT) has an important influence on the WRS distribution of welded joints. Shen et al. [5] studied the effect of SSPT on the WRS of EH690 high-strength steel during welding. The results show that the accuracy of the analysis residual stress can be greatly improved by considering the SSPT, since the distribution form and amplitude of WRS can be changed. In practical manufacturing scenarios, the WRS caused by SSPT is difficult to be measured by experiments, and as a result, its impact on stress evolution is often overlooked. While the aforementioned studies highlight the significance of SSPT in WRS formation, the contribution of transformation plasticity (TP) remains inadequately quantified.

During phase transformation in materials, the application of a load below the elastic limit of a softer phase, such as austenite, can induce irreversible deformation—this phenomenon is known as TP. In recent years, TP has attracted considerable attention as a distinct form of irreversible deformation, separate from classical plasticity, garnering interest across the fields of materials science, mechanics, and physics. Hu et al. [6] demonstrated that TP can effectively reduce the overall level of welding-induced residual stress in P91 steel. Empirical evidence further indicates that accurately accounting for TP is crucial for improving the precision of residual stress predictions in numerical simulations [7,8,9]. However, most current studies rely on approximate TP coefficients derived from other steels [10,11,12]. Given the high alloy content and superior hardenability of 30MnCrNiMo steel, its TP behavior may significantly diverge from that of conventional steels [13]. Moreover, the specific influence of TP on the residual stress of 30MnCrNiMo ultra-high-strength steel remains unreported.

To validate numerical simulation accuracy for residual stress in composite welds, the cosα method in X-ray diffraction was employed for experimental measurement—an approach whose reliability has been extensively confirmed [14,15]. The TP coefficient and phase transformation kinetics were calibrated through thermal simulation testing. A multi-field coupling model was developed, and four simulation cases were designed. Comparing the simulation and experimental results, the influence of TP on the stress distribution is revealed. The research results establish a mechanical database of 30MnCrNiMo steel, which provides a basis for quantification of the WRS and welding process of armored steel welded structural parts.

## 2. Experimental Procedures

The laser–MAG hybrid welding process (HLAW) combines a laser heat source and a metal active gas arc welding (MAG) heat source, which overcomes the limitations of a single heat source through a synergistic effect [16]. The advantages of this process arise from the synergistic effects of the laser’s deep penetration capability and the MAG arc’s superior filler deposition: (1) the high energy density of the laser enables deep penetration welding, thereby reducing the need for multi-pass welding; (2) the MAG arc enhances gap-bridging ability and improves process stability; (3) the low heat input (energy per unit length) significantly narrows the Heat-Affected Zone (HAZ), minimizing stress concentrations and the risk of failure due to microstructural degradation in the HAZ [17,18]. These mechanisms are particularly advantageous in welding high-strength armored steel, where the HLAW process achieves low residual stress, minimal deformation, and excellent crack resistance [19], making it an ideal platform for subsequent WRS investigations.

To verify the application effect of the HLAW process in 30 MnCrNiMo armor steels, a flat butt welding design (sample size: 250 mm × 100 mm × 8 mm) was adopted in this study. The base metal is low-alloy high-strength steel 30MnCrNiMo, which is welded using matching stainless steel filler wire H1Cr21Ni10Mn7 (composition given in Table 1). This stainless steel filler reduces the cracking susceptibility of the welded joint, enhancing its reliability and structural integrity. Moreover, it can eliminate the need for preheating and post-weld heat treatment, thereby improving the process weldability of the steel. The welding process parameters were as follows: laser power, 3900 W; welding speed, 13.3 mm/s; defocusing amount/filament spacing, 3 mm; welding current, 212 A; voltage, 22.8 V; and shielding gas, 20% CO_2_ + 80% Ar (flow rate, 20 L/min). A single-sided welding process was adopted to allow free shrinkage deformation of the sample. To monitor the thermal cycle characteristics, two K-type thermocouples were arranged at different positions in the middle section of the upper surface.

The welding residual stress was measured using a μ-360s X-ray residual stress tester developed by Pulstec, Japan. The stress determination method is based on two-dimensional detection of the Debye ring and subsequent image data analysis. For plane stress analysis, measurement can be performed using only a single Debye ring, eliminating the need for an X-ray incident angle adjustment or a diffraction beam positioning instrument. The measurement parameters of the residual stress tester are presented in Table 2.

## 3. Determination of the Transformation Plasticity Coefficient

### 3.1. TP Test

**Figure 1:**
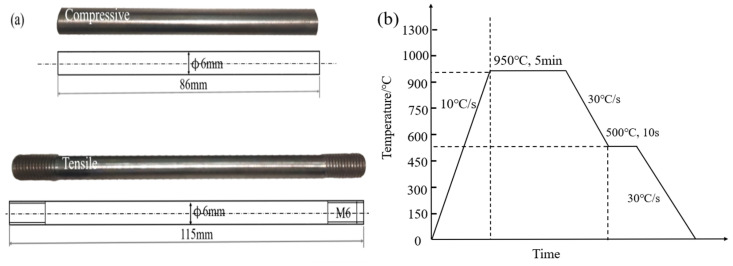
Experimental dilation for 30MnCrNiMo steel: (**a**) specimen geometry and (**b**) experimental scheme.

The TP experiments were carried out on a Gleeble 3500 thermal simulation machine; a sample is shown in Figure 1a. When the effect of TP on stress was studied, the radial strain change was measured by applying an axial load and a thermal dilatometer. To avoid the interference of stress on the martensitic transformation point, the loading temperature was set at 500 °C and maintained for 10 s to achieve a stable temperature load. A controlled axial tensile and compressive stress gradient was implemented to ensure the mechanical integrity of the specimen during loading. Throughout the loading process, the axial stress was maintained below the yield strength of the material at 500 °C, thereby preventing plastic deformation. The peak heating temperature of the specimen is higher than the full austenitization temperature, resulting in an austenitic microstructure when cooled to 500 °C. As reported by Byun et al. [20], the yield strength of austenite at this temperature is 117 MPa. Accordingly, the stress gradient was configured within the range of 0 to ±100 MPa. Considering the critical cooling rate required for complete martensitic transformation, a rate of 30 °C/s was applied. The test procedure is illustrated in Figure 1b.

### 3.2. Results of the Phase Transformation Plasticity Test

**Figure 2:**
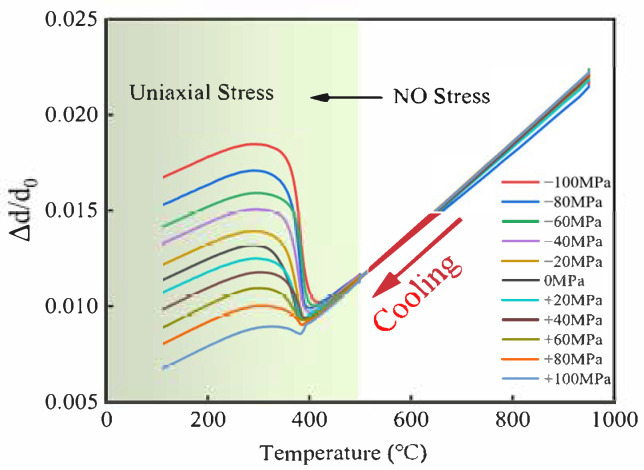
Experimental dilation curves under different stresses.

Figure 2 shows the radial strain thermal expansion curves under applied axial tensile and compressive stresses. Prior to the application of stress, the thermal expansion behavior is linear with respect to temperature, as no phase transformation occurs in the supercooled austenite. Upon reaching 500 °C, the application of axial stress introduces a slight increase in elastic strain relative to the unstressed condition. As the temperature falls below the Ms, martensitic transformation initiates, marking a deviation from the linear expansion trend. In addition to the thermal strain and the volumetric strain of the phase transformation, the stress also causes plastic strain, and these strains change significantly with increasing martensitic transformation. After the phase transition, the two ends of the expansion curve tend to be parallel. The figure shows that the radial expansion caused by compressive stress is greater than the free expansion (0 MPa), whereas the tensile stress has the opposite effect. The final strain is affected by the combined action of thermal strain, phase transformation volumetric strain and phase transformation plastic strain, and the phase transformation plasticity has a significant effect on the total strain.

### 3.3. Calculation of the Transformation Plasticity Coefficient

Under the influence of applied stress, the thermal expansion behavior comprises five components: thermal strain, elastic strain, phase transformation volumetric strain, classical plastic strain, and phase transformation plastic strain [21]. As the applied stress remains below the material’s yield strength, classical plastic strain is negligible and can be considered zero. Therefore, the constitutive equation, excluding classical plasticity, is expressed as

(1) $$\varepsilon^{\sigma }=\varepsilon^{E}+\varepsilon^{Tp}+\varepsilon^{Th}+\varepsilon^{Vol}$$

where $\varepsilon^{\sigma }$ is the total strain, $\varepsilon^{Th}$ is the thermal strain, $\varepsilon^{Vol}$ is the volumetric strain of the phase transformation, $\varepsilon^{E}$ is the elastic strain, and $\varepsilon^{Tp}$ is the plastic strain of the phase transformation.

The mechanical analysis uses the generalized Hooke law related to Young’s modulus to calculate the elastic strain. The calculation results of the radial elastic strain are as follows:

(2) $$\varepsilon^{E}=-\mu (\sigma /E)$$

where μ is Poisson’s ratio, μ=0.3, σ is the size of the stress, and *E* is Young’s modulus.

Since the sum of $\varepsilon^{Th}$ and $\varepsilon^{Vol}$ is unchanged under different stress states, it can be obtained from the thermal expansion curve under stress-free conditions. $\varepsilon_{rr}^{Tp}$ is the radial strain obtained by measurement. Therefore, according to the principle of volume invariance [22], the axial phase change plastic strain $\varepsilon_{zz}^{Tp}$ can be expressed as

(3) $$\varepsilon_{zz}^{Tp}=-\varepsilon_{rr}^{Tp}/0.5=K\cdot \sigma_{zz}\cdot f(z)$$

where $\sigma_{zz}$ is the uniaxial stress and *f*(*z*) = 1 when the martensitic transformation is completed.

**Figure 3:**
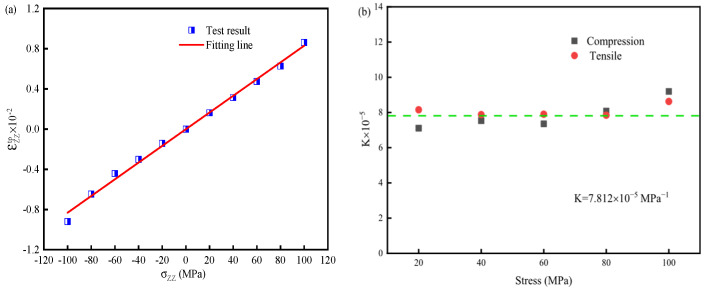
Calibration of TP: (**a**) Curve of $\varepsilon_{zz}^{Tp}$ -$\sigma_{zz}$ (**b**) Coefficient K and applied stresses.

The $\varepsilon_{zz}^{Tp}$-$\sigma_{zz}$ curve is shown in Figure 3a. Figure 3b illustrates the relationship between $\sigma_{zz}$ and *K* under different applied loads, showing that the stress magnitude has a minimal effect on the value of *K*. Therefore, the TP expression for 30MnCrNiMo steel is as follows:

(4) $$\varepsilon^{Tp}\;=\;7.812\;\times \;10^{-5}\cdot z\cdot (2\;-\;z)\cdot \bar{\sigma }$$

### 3.4. Measurement of Weld Residual Stress

**Figure 4:**
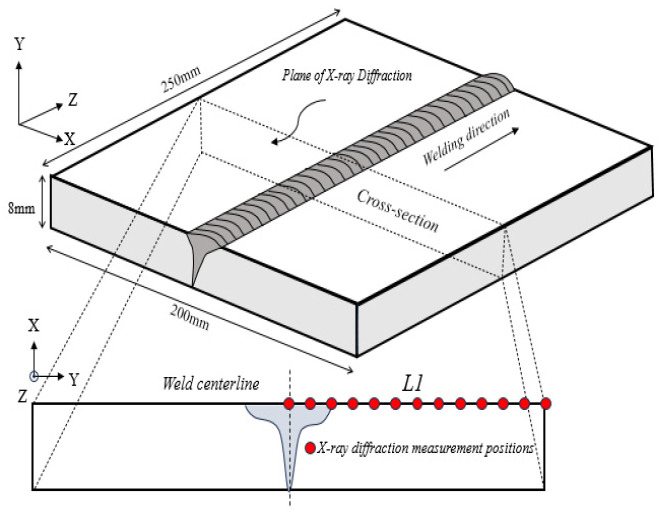
Schematic diagram of the welding experiment.

Following the completion of the test, the WRS of the welded specimen was measured using a μ-X360 s X-ray stress measuring instrument manufactured by Pulstec, Hamamatsu, Japan. This device operates based on the cosα method, which analyzes the diffraction Debye ring to determine residual stress. The cosα method represents a new method for measuring stress [23]. Since the surface layer of the weldment is in biaxial stress state and the stress in the thickness direction is zero, the problem can be simplified to a plane stress condition within the penetration depth of XRD. The measurement locations are illustrated in Figure 4.

## 4. Numerical Simulation

### 4.1. Finite Element Model

**Figure 5:**
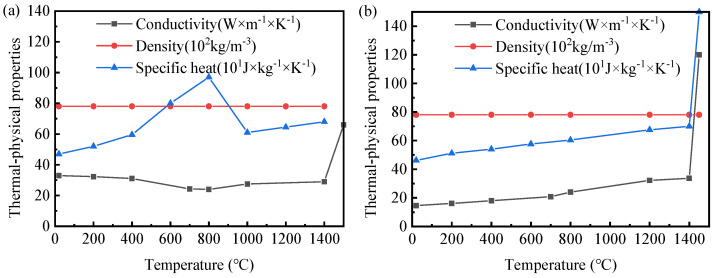
Thermal physics of the material. (**a**) Base metal. (**b**) Filler metal.

**Figure 6:**
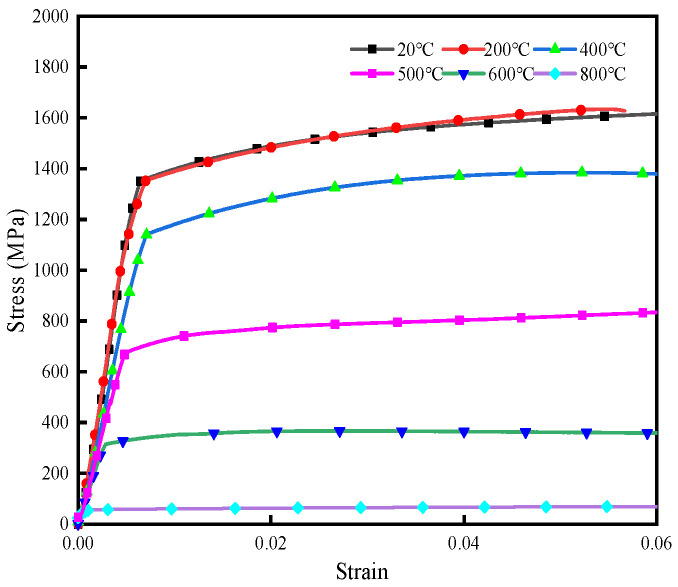
The stress–strain curves of 30 Mn Cr Ni Mo at different temperatures.

Welding is a complex process that involves the coupling of multiple physical fields, including the temperature field, stress–strain field, and microstructural field. Therefore, this paper uses the thermal–metallurgical–mechanical coupling model for calculation. As no existing phase transformation model for 30MnCrNiMo steel has been reported, a coupled model was developed and computed for this study. Due to the unavailability of high-temperature material data, thermal–physical properties varying with temperature were obtained using the JMatPro material simulation software (Version 7.0) [24], as illustrated in Figure 5. The intermediate value was obtained by linear interpolation according to the adjacent temperature range. The thermal expansion coefficients for different phases were calculated using the tangent method [25] and are presented in Table 3. In this study, the stress–strain behavior of 30MnCrNiMo steel at both room and elevated temperatures was characterized through tensile testing, performed at a loading rate of 2 mm/min. The resulting true stress–strain curve of the initial phase is shown in Figure 6. Mechanical property parameters for the filler material were adopted from the austenite phase data reported in the literature [26].

**Figure 7:**
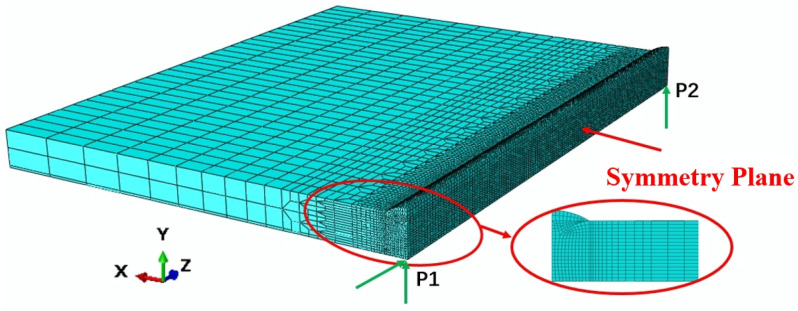
Schematic diagram of the FEM and restraint conditions.

The Finite Element Model (FEM) used a model that is consistent with the actual size of the welded plate. To balance the relationship between the calculation time and accuracy, the temperature gradient in the welded joint area was large, and a finer mesh was used, while the base metal area was divided into excessive mesh and sparse mesh. As shown in Figure 7, the total numbers of elements and nodes used for FEM analysis were the same, which were 57,381 and 62,532, respectively. The mesh convergence criterion was based on the peak temperature becoming independent of further increases in element count [27]. Additionally, it was ensured that the heat source covered at least four nodes [15]. An eight-node linear diffusion heat transfer element (DC3D8) was employed for thermal analysis, while the C3D8 element was used for mechanical analysis. Mechanical boundary conditions were applied to constrain rigid body displacements and rotations, as illustrated in Figure 7. The thermal–metallurgical–mechanical model was solved using the finite element software ABAQUS/Standard (Version 2020), utilizing a sequential coupling approach. In this method, the temperature distribution of the welded specimen was first computed and subsequently applied as the initial condition for the stress field analysis, thereby achieving the coupled simulation.

### 4.2. Calculation of the Temperature Field and Microstructure Field

The heat source application was implemented using the DFLUX subroutine, which enables the definition of a composite heat source with a specified heat flux distribution. Accurate calibration of the heat source is critical for ensuring the reliability of both metallographic and mechanical analyses. Considering the funnel-shaped cross-section of the molten pool characteristic of HLAW, the total heat input was modeled using a composite heat source comprising a double ellipsoidal and cylindrical distribution [28].

Heat exchange between the weldment and the surrounding environment occurs primarily through convection and radiation at the temperature boundaries. It is defined by Equations (5) and (6) [29].

(5) $$q_{conv}=h_{conv}(T_{s}-T_{0})$$

where $h_{conv}$ is the convective heat transfer coefficient, and the value is 25 W/(m^2^∙K) [30]; $T_{0}$ is ambient temperature of the sample and is set to 20 °C; $T_{s}$ is the instantaneous surface temperature of the welded specimen; and $T_{abs}$ is absolute zero.

(6) $$q_{rad}=\varepsilon \sigma \left[{\left(T_{s}-T_{abs}\right)}^{4}-{\left(T_{0}-T_{abs}\right)}^{4}\right]$$

where ε is the surface emissivity coefficient, which is typically 0.8 for steel [31], and *σ* is the Stefan–Boltzmann constant, which is 5.67 × 10^−8^ J∙K^−4^∙m^−2^⋅s^−1^.

**Figure 8:**
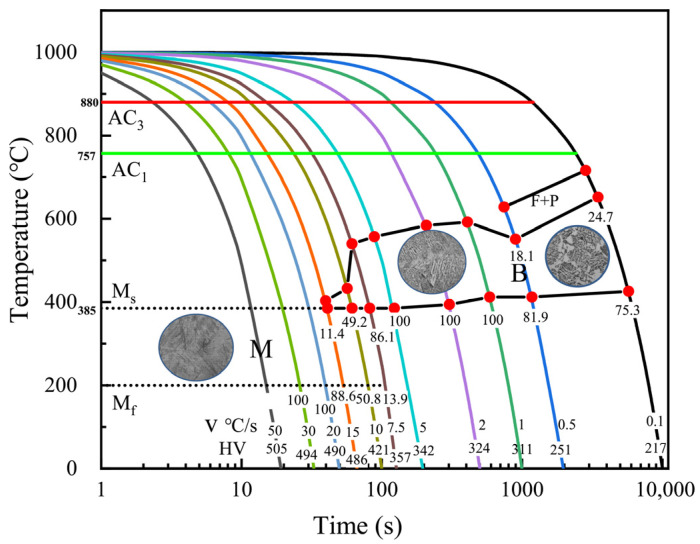
The experimental continuous transformation curves of the CCT diagram.

During the hybrid welding process, heat input induces a high-temperature thermal cycle in the welded joint area, leading to microstructural transformations. To simulate these microstructural changes under varying thermal cycles, thermal expansion curves at different cooling rates were measured using a thermal simulation testing machine. Figure 8 is the continuous cooling transformation (CCT) curve in the cooling rate range of 0.5 °C/s~50 °C/s. The results indicate that when the cooling rate exceeds 20 °C/s, austenite undergoes complete transformation into martensite.

**Figure 9:**
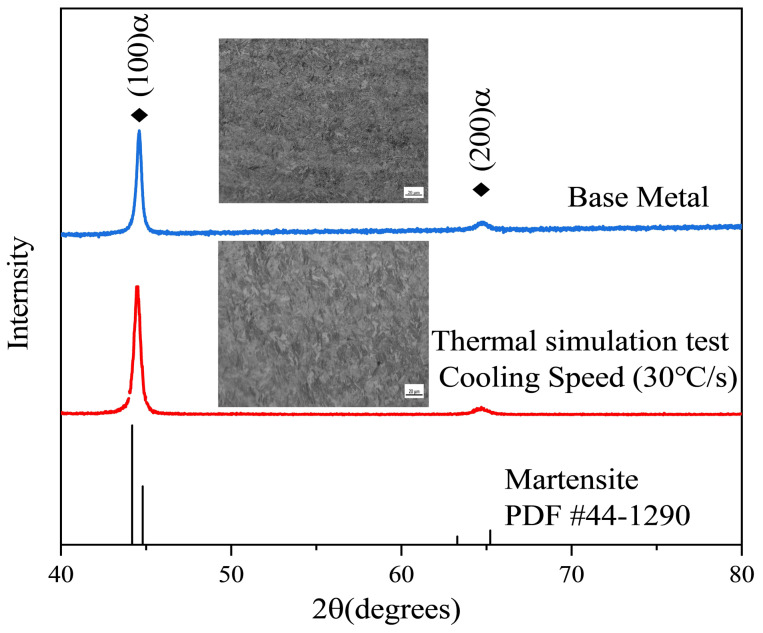
Metallurgical results of XRD diffraction analysis of base metal and thermal simulation test sample.

Phase analysis of the retained austenite was carried out via X-ray diffraction to determine the microstructure of the retained austenite after the welding process. A copper target X-ray with a wavelength of 0.15 nm was used in the detection process. The scanning angle was 40°~80°, and the scanning speed was 6°/min. The results are shown in Figure 9. The results show that the base metal and the samples subjected to thermal simulation at a cooling rate of 30 °C/s are all martensite, and the austenite is not detected. This may be because the content of retained austenite is less than 5%, which is lower than the detection limit of the instrument, and no diffraction peak is generated. Therefore, the retained austenite phase can be neglected in the model.

### 4.3. Stress and Strain Calculation

In the welding simulation, the stress prediction can be decomposed into five parts of the total strain. Equation (1) is transformed into an incremental form, considering the increase in plastic strain.

(7) $${d\varepsilon }^{Totol}=d\varepsilon^{E}+{d\varepsilon }^{P}+{d\varepsilon }^{Th}+{d\varepsilon }^{Vol}+{d\varepsilon }^{Tp}$$

where $d\varepsilon^{E},\;\;{d\varepsilon }^{P},\;\;{d\varepsilon }^{Th},\;\;{d\varepsilon }^{Vol}\;and\;\;{d\varepsilon }^{Tp}$ are the increments of elastic strain, plastic strain, thermal strain, phase transformation volumetric strain and phase transformation plastic strain, respectively.

To investigate the effect of TP on martensitic transformation in 30MnCrNiMo steel, the kinetics of martensitic transformation under varying external loads was examined. The transformation of undercooled austenite to martensite during cooling was calculated via the K-M equation [32].

(8) $$f_{M}=1-\exp\left(-\alpha \left(M_{s}-T\right)\right)(T\leq M_{s})$$

where $M_{s}$ is the starting temperature of martensitic transformation; *α* is a constant related to the material composition; and *T* is the current temperature of the sample.

**Figure 10:**
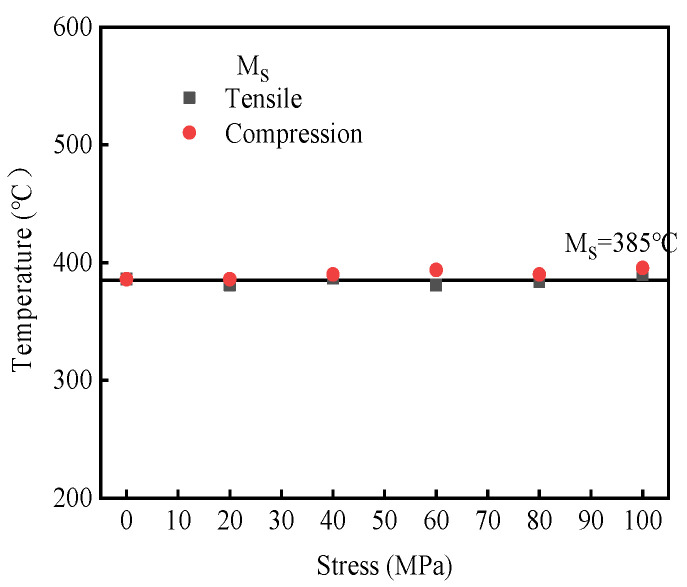
Starting temperature of martensite transformation kinetics in 30MnCrNiMo steel.

The martensitic transformation start temperature ($M_{s}$) of the thermal expansion curve was calibrated via the tangent method under different applied loads, as shown in Figure 10. The initial temperature of martensitic transformation under compressive stress is slightly higher than that observed under tensile stress; however, the overall variation is minimal and shows no clear correlation with the absolute value of the applied stress. Through linear fitting, the martensite starting temperature can be obtained as $M_{s}$ = 385 °C.

Take any temperature, $T_{i}$, in Figure 2, corresponding to a radial expansion $\Delta d_{i}^{\sigma }$. At this time, $d_{i}^{\sigma }/d_{0}$ includes three parts: volumetric expansion caused by microstructural changes ${\left(\Delta d∕d_{0}\right)}^{Vol}$, expansion caused by phase transformation plasticity ${\left(\Delta d∕d_{0}\right)}^{Tp}$ and elastic deformation ${\left(\Delta d∕d_{0}\right)}^{E}$. That is,

(9) $$\Delta d_{i}^{\sigma }/d_{0}={\left(\Delta d∕d_{0}\right)}^{Vol}+{\left(\Delta d∕d_{0}\right)}^{Tp}+{\left(\Delta d∕d_{0}\right)}^{E}$$

where

(10) $${\left(\Delta d∕d_{0}\right)}^{Vol}=\beta_{M}^{0}f_{M}+\alpha_{M}T_{i}f_{M}+(1-f_{M})\alpha_{A}T_{i}$$

(11) $${\left(\Delta d∕d_{0}\right)}^{Tp}=-0.5\cdot K\cdot \sigma \cdot f_{M}(2-f_{M})$$

(12) $${\left(\Delta d∕d_{0}\right)}^{E}=-0.3\cdot \sigma /E$$

**Figure 11:**
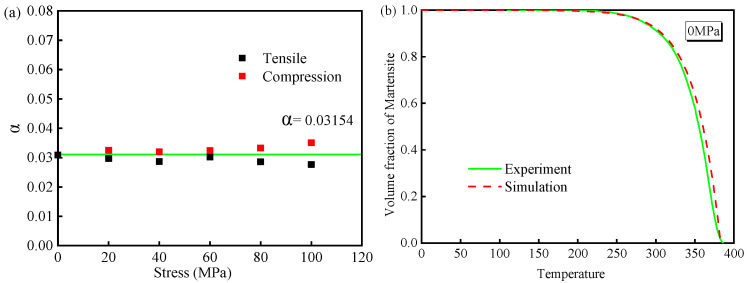
Kinetic parameters of 30MnCrNiMo steel: (**a**) *α* values of the material constant and (**b**) volume fraction vs. transformation temperature of martensite.

Substituting Equations (10)–(12) into Equation (9), we obtain

(13) $$0.5\cdot K\sigma f_{M}^{2}{+(\beta }_{M}^{0}+\alpha_{M}T_{i}-\alpha_{A}T_{i}-K\sigma )f_{M}+\alpha_{A}T_{i}-0.3\cdot \sigma /E-\Delta d_{i}^{\sigma }/d_{0}=0$$

where ${\;\beta }_{M}^{0}$ is the volumetric strain generated by the microstructure transformation without stress, *K* is the *TP* coefficient, $f_{M}$ is the volume fraction of martensite at any time, σ is the applied stress, E is the elastic modulus, and $\alpha_{A\;}and\;\alpha_{M}$ are the linear expansion coefficients of austenite and martensite, respectively. Equation (13) is a quadratic equation in terms of $f_{M}$, and the material constants in Equation (8) are determined based on the condition that the calculated results are positive. The material constants under different stress conditions are shown in Figure 11a. Although there are slight fluctuations in the values, the overall variation is minimal, and they can be approximated as constant. The phase transformation kinetic parameter α for 30MnCrNiMo steel is $3.15\times 10^{-2}$.

The simulation curve of martensite content is obtained by substituting α and Ms into Equation (8). Taking the martensitic transformation volume curve at 0 MPa as an example, the simulated curve shown in Figure 11b is quite similar to the experimental curve, indicating that the simulation accurately reflects the martensitic transformation kinetics of 30MnCrNiMo steel. The applied stress has no significant influence.

There are usually two mechanisms to explain the TP: the Magee mechanism and Greenwood–Johnson mechanism. The Greenwood–Johnson mechanism can explain most cases of small stress. Owing to the low stress level in the welding and heat treatment process, the Greenwood–Johnson mechanism is selected for this work. The general formula for the TP strain, as derived from this mechanism [33], is as follows:

(14) $$d\varepsilon^{Tp}=\frac{3}{2}K_{Tp}\sigma^{\prime }f^{\prime }(z)dz$$

where $\sigma^{\prime }$ is the deviatoric stress and *f*(*z*) is the evolution function of the TP.

### 4.4. Case Design

In order to study the effect of TP on the WRS of 30MnCrNiMo steel, four calculation results under different SSPT conditions are listed in Table 4. Case 1 does not consider SSPT. Case 2 increases the yield strength of different phases compared with Case 1. Case 3 further considers the influence of the phase change volumetric strain, whereas Case 4 considers the influences of the phase change volumetric strain and phase change plasticity at the same time.

## 5. Results and Discussion

### 5.1. Verification of the Temperature Field

**Figure 12:**
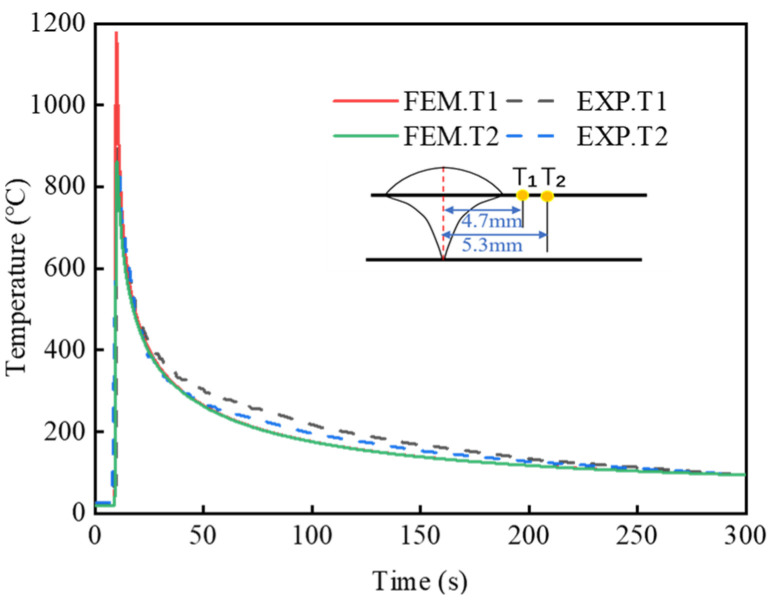
Thermal cycle curves.

In the sequentially coupled analysis, the results of the thermal analysis directly determine the accuracy of subsequent metallurgical and mechanical analyses. Based on the temperature history curve recorded during the welding test, calibration of the heat source model is essential. Thermocouples T1 and T2 are positioned at different distances from the weld center to monitor temperature changes. The temperature history recorded by thermocouples T1 and T2 is used to validate the accuracy of the thermal simulation results [34]. Figure 12 presents the temperature histories obtained from both experimental measurements and finite element simulations. The comparison demonstrates that the transient temperature distribution in the 30MnCrNiMo high-strength steel butt joint predicted by the simulation closely matches the experimental data, confirming the accuracy of the thermal analysis model.

### 5.2. Stress Evolution Analysis

**Figure 13:**
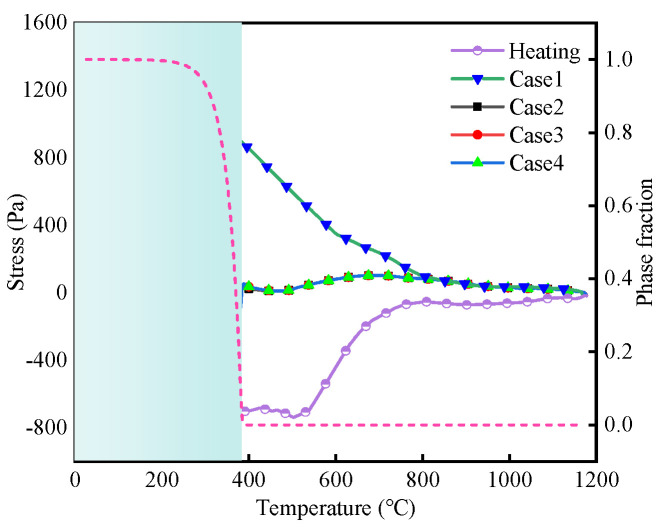
Predicted stress evolution and martensite fraction evolution.

A check point was defined on the upper surface of the FEM, 4.7 mm away from the weld centerline. The peak temperature at this point reaches 1175 °C. Positioned within the HAZ, this point effectively captures the stress evolution associated with complete microstructural transformation throughout the process. The stress evolution curve and martensite transformation curve during the welding process are shown in Figure 13. In Case 1, where SSPT is not considered, the tensile stress increases monotonically as the temperature decreases, ultimately reaching the material’s yield strength at room temperature. In Cases 2 through 4, the stress evolution in the austenite phase during both heating and cooling phases shows similar trends. Owing to the low flow stress of austenite, the tensile stress fluctuates within a narrow range. In Case 2, a rapid increase in tensile stress is observed following the onset of martensite formation, primarily due to the influence of phase-dependent yield strength. The yield strength of undercooled austenite in Case 2 is significantly lower than that of the initial phase, leading to a slower stress increase in the temperature range above the martensite start temperature. In Case 3, where volumetric strain associated with phase transformation is considered, the tensile stress does not continue to rise but instead decreases, eventually transitioning into compressive stress. An inflection point appears at 292 °C, beyond which the stress begins to increase again. By analyzing the martensite volume fraction curve, it is evident that the initial rate of martensitic transformation is high, and the associated volumetric strain dominates, counteracting the effects of increasing flow stress and thermal contraction during the cooling phase. After the temperature is reduced to the inflection point, the martensitic transformation rate is reduced, the heat shrinkage strain is dominant, and the stress curve shows an increasing trend. For Cases 3 and 4, some similar trends are observed during the martensitic transformation. In Case 4, after considering the phase transformation plasticity, when the martensitic transformation begins, the phase transformation plasticity increases with increasing martensitic transformation fraction. Because the martensitic transformation is an instantaneous shear process, the phase transformation plasticity increases rapidly with decreasing temperature. When the volume fraction of martensite ranges from 0.12 to 0.45, phase transformation plasticity becomes the dominant factor, resulting in a turning point on the stress curve in the opposite direction. As the temperature continues to decrease, phase transformation volumetric strain gradually becomes dominant. Throughout the entire transformation process, the influence of TP offsets most of the volumetric strain.

**Figure 14:**
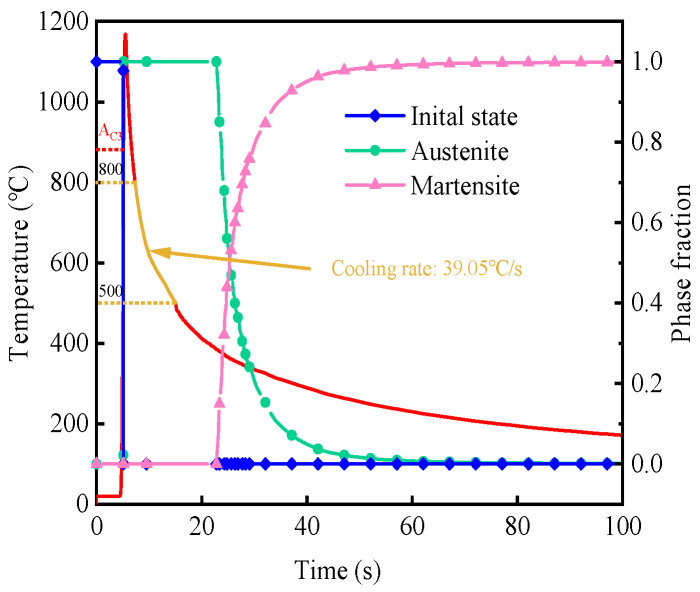
Thermal cycle and microconstituent fraction curves at the point.

Figure 14 illustrates the thermal cycle curve and microstructural transformation at the feature points within the HAZ of the welded joint. When the peak temperature exceeds the AC3 temperature, the initial phase is fully transformed into austenite. The transformation of austenite into other phases during cooling is governed by the cooling time (t8/5) in the 800–500 °C range [35]. Based on the thermal cycle curve, the cooling rate in the t8/5 temperature range during the cooling phase is 39 °C/s, which exceeds the critical cooling rate for martensitic transformation, previously determined as 20 °C/s through thermal simulation testing. Therefore, during the cooling process, only austenite and martensite phases are present, and the austenite content remains unchanged until the onset of martensitic transformation. Ultimately, in the corresponding transformation temperature zone, austenite is fully transformed into martensite at room temperature.

**Figure 15:**
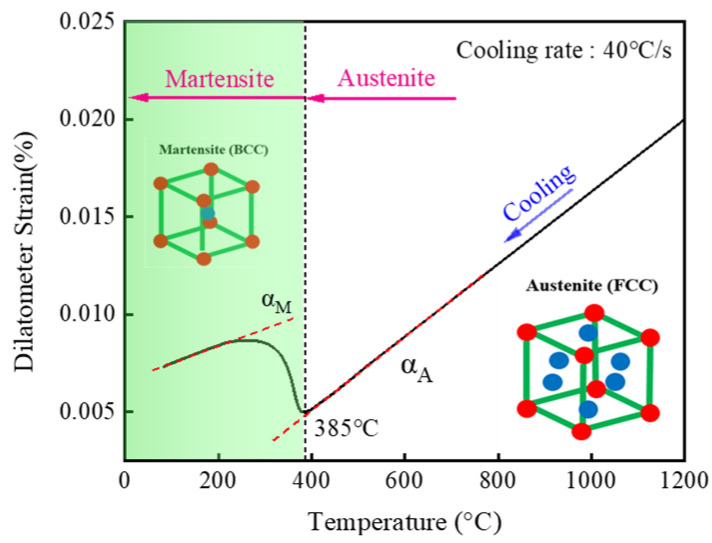
Dilatometric curve used to simulate the actual cooling rate.

A thermal expansion test was conducted using the thermal cycle curve depicted in Figure 14 to validate the phase transformation calculation during cooling. The results are shown in Figure 15. The sample was heated to 1200 °C and kept warm to make it fully austenitized before it was rapidly cooled. Prior to 385 °C, the expansion curve remains linear, indicating that the austenite phase does not transform into other phases. As the temperature decreases further, a noticeable change in the expansion curve is observed. The starting and ending temperatures of the phase transition obtained by the tangent method are consistent with the temperature of the CCT curve. Moreover, the diagram shown in Figure 9 further verifies that the phase transformation result is martensite, which is in good agreement with the finite element calculation result.

### 5.3. Welding Residual Stress

The mutual transformation between martensite and austenite during welding is the root cause of the influence of SSPT on the simulation results of the stress field, which is caused mainly by three factors: the material yield strength caused by phase transformation and the volumetric strain and plastic strain produced by phase transformation. Therefore, an in-depth analysis of the austenite/martensite transformation behavior during the welding heating/cooling process is highly important for understanding the influence of the SSPT behavior on the stress field.

**Figure 16:**
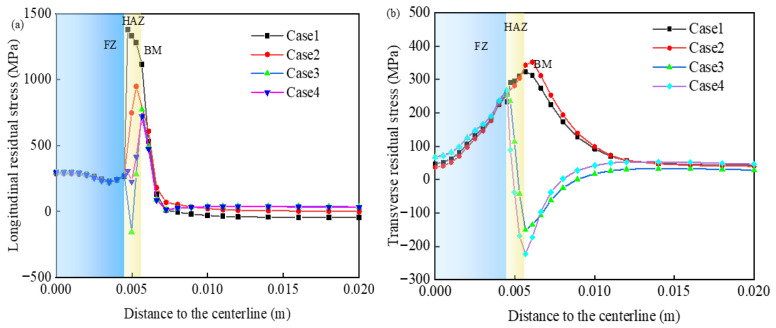
The calculation results of WRS distributions: (**a**) longitudinal residual stress and (**b**) transverse residual stress.

The longitudinal and transverse stress components of the WRS are shown in Figure 16. Figure 16 shows that the difference between these conditions is negligible in the base metal zone of the weld and the fusion zone in the weld center, but the difference near the HAZ is quite large. Because the weld uses low-strength matching stainless steel wire as the filler metal, the stress in the weld center is low. The drastic change in the HAZ indicates that SSPT affects mainly the WRS inside and near the HAZ.

From the curves corresponding to Case 1 and Case 2 in Figure 16a, it can be observed that the maximum longitudinal residual tensile stress in Case 1, which does not consider SSPT, is 1471 MPa. This value is close to the yield strength of the material at room temperature, and the maximum tensile stress occurs near the fusion line, representing the most conservative prediction. In comparison, after accounting for the change in yield strength due to phase transformation in Case 2, the peak longitudinal tensile stress decreases from 1471 MPa to 947 MPa. Additionally, the peak location of longitudinal stress in the HAZ shifts toward the base metal, although this has minimal effect on the transverse residual stress, as shown in Figure 16b.

The longitudinal residual stress distribution in Cases 3 and 4, which incorporate transformation volumetric strain, exhibits an opposite trend near the HAZ center compared to Cases 1 and 2. In the absence of volumetric strain (Cases 1 and 2), the longitudinal residual stress initially increases and then decreases toward the weld center. Conversely, in the presence of volumetric strain (Cases 3 and 4), the longitudinal stress displays two inflection points as it approaches the weld center. The transverse stress distribution in Figure 16b shows a similar turning behavior in the HAZ, where the transverse stress transitions from tensile (in Cases 1 and 2) to compressive (in Cases 3 and 4).

The corresponding curves for Cases 2 and 3 demonstrate that, following modifications to the thermal expansion coefficient and the inclusion of volumetric strain induced by phase transformation, the WRSs in all directions undergo significant changes. Notably, the longitudinal residual stress shifts from tensile to compressive. The peak compressive stress in the HAZ is about −156 MPa, exhibiting a sharp transition. Following the martensitic transformation, the absolute value of the volumetric strain increment exceeds that of the thermal strain increment, leading to a gradual decrease in longitudinal tensile stress, which eventually shifts from a tensile to a compressive state. The transverse residual stress is predominantly tensile. At the adjacent position of the interface between the HAZ and the base metal, the transverse stress transits from tension to compression, and the peak stress is −150 MPa.

The curves corresponding to conditions 3 and 4 show that in the HAZ, the peak tensile stress of the longitudinal residual stress is reduced by about 51 MPa, and the compressive stress disappears. After considering the phase transformation plastic strain, the transverse residual stress is reduced, and the peak value of compressive stress is reduced from −150 MPa to −223 MPa. Compared with those in Case 3, the longitudinal stresses in the HAZ in Case 4 decrease, the transverse stresses increase, and the stress changes are greater than those in A335 steel [10] and EH40 steel [12]. It shows that different steel grades have different TP coefficients and different effects on WRS.

In summary, when the SSPT behavior is taken into account, the residual stress exhibits a significantly altered distribution pattern and magnitude in all directions. Among the influencing factors, the phase transformation volumetric strain has the most substantial impact, followed by the transformation plastic strain, while the effect of yield strength variation is comparatively minimal.

**Figure 17:**
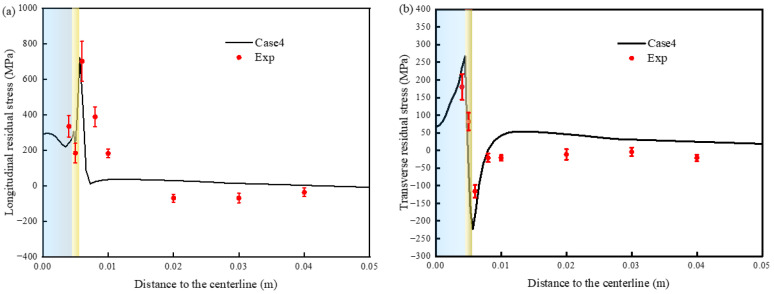
Comparison between the experimental and simulation results: (**a**) longitudinal residual stress and (**b**) transverse residual stress.

According to the results shown in Figure 17, the WRS distribution in Case 4 is closest to the trend and characteristics of the experimental data, with an average error of 8.8%. A slight discrepancy exists between the simulated values and the stress measured via XRD, which may be attributed to initial residual stresses induced during processing prior to the welding experiment [36]. Within the HAZ, over a width of about 1.5 mm, the longitudinal tensile stress exhibits a sharp increase from 225 MPa to 721 MPa. The transverse stress demonstrates a similar magnitude of fluctuation but follows an opposite trend. Although Case 4 slightly overestimates the peak longitudinal tensile stress, the simulated value remains close to the experimental measurement. Notably, both reversal trends in the HAZ are effectively captured. Therefore, the deviation between the experimental and simulated stress distribution curves is considered acceptable.

## 6. Conclusions

The experimental and numerical simulation results demonstrate that TP plays a critical role in the evolution of WRS during HLAW of 30MnCrNiMo armor steel. The main findings are summarized as follows:

(1) The TP coefficient (7.81 × 10^−5^ MPa^−1^) has no obvious dependence on the applied stress gradient (0–±100 MPa), which verifies the applicability of the Greenwood–Johnson mechanism in high-strength steel.
(2) In the range of 0.12–0.45 volume fractions of martensite, the transformation plastic strain dominates the evolution of WRS, whereas the volumetric strain dominates in other stages of phase transformation.
(3) The incorporation of the phase transformation plasticity (TP) effect results in a reduction in the longitudinal peak compressive stress by about 50 MPa, and the compressive stress is simultaneously eliminated. This indicates that the influence of TP on the WRS distribution is significant and cannot be overlooked.
(4) The TP coefficient calibration method proposed in this paper can provide a scientific basis for accurately predicting the WRS of high-strength steel.

## Figures and Tables

**Table 1:** Chemical composition of 30MnCrNiMo steel and filler wire H1Cr21Ni10Mn7 (wt %).

Materials	C	Si	Mn	P	Cr	Ni	Mo	V	Nb	Fe
30MnCrNiMo	0.30	0.33	1.01	0.009	1.04	1.30	0.43	0.19	0.033	Bal.
H1Cr21Ni10Mn7	0.087	0.40	6.75	0.026	21.10	10.3	–	–	–	Bal.

**Table 2:** Parameters used for stress measurement by XRD method.

Plane	Target	Wavelength (Å)	Voltage (kV)	Current (mA)	Bragg Angle	Young’s Modulus (GPa)	Spatial Resolution (mm)
211	Cr	2.866	30	1.5	156.4°	224	1

**Table 3:** Thermal expansion coefficient and total volume strain of different phases.

Microconstituent	Thermal Expansion Coefficient (°C^−1^)	Volumetric Change Strain Increment
Initial stare (I)	1.1 × 10^−5^	/
Austenite (A)	2.3 × 10^−5^	2.88 × 10^−3^ (I-A)
Martensite (M)	1.2 × 10^−5^	5.35 × 10^−3^ (A-M)

**Table 4:** Cases examined.

Cases	Yield Stress Change	Volumetric Strain	Transformation Plasticity
Case 1	NO	NO	NO
Case 2	YES	NO	NO
Case 3	YES	YES	NO
Case 4	YES	YES	YES

## Data Availability

The original contributions presented in this study are included in the article. Further inquiries can be directed to the corresponding author.
